# NMR Metabolomics of Primary Ovarian Cancer Cells in Comparison to Established Cisplatin-Resistant and -Sensitive Cell Lines

**DOI:** 10.3390/cells13080661

**Published:** 2024-04-09

**Authors:** Veronica Ghini, Flavia Sorbi, Massimiliano Fambrini, Francesca Magherini

**Affiliations:** 1 Department of Chemistry “Ugo Schiff”, University of Florence, 50019 Sesto Fiorentino, Italy; 2 Magnetic Resonance Center (CERM), University of Florence, 50019 Sesto Fiorentino, Italy; 3 Department of Experimental and Clinical Biomedical Sciences “Mario Serio”, University of Florence, 50134 Florence, Italy; flavia.sorbi@unifi.it (F.S.); massimiliano.fambrini@unifi.it (M.F.)

**Keywords:** ovarian cancer, cell lines, NMR, metabolomics, primary cells

## Abstract

Cancer cell lines are frequently used in metabolomics, such as in vitro tumor models. In particular, A2780 cells are commonly used as a model for ovarian cancer to evaluate the effects of drug treatment. Here, we compare the NMR metabolomics profiles of A2780 and cisplatin-resistant A2780 cells with those of cells derived from 10 patients with high-grade serous ovarian carcinoma (collected during primary cytoreduction before any chemotherapeutic treatment). Our analysis reveals a substantial similarity among all primary cells but significant differences between them and both A2780 and cisplatin-resistant A2780 cells. Notably, the patient-derived cells are closer to the resistant A2780 cells when considering the exo-metabolome, whereas they are essentially equidistant from A2780 and A2780-resistant cells in terms of the endo-metabolome. This behavior results from dissimilarities in the levels of several metabolites attributable to the differential modulation of underlying biochemical pathways. The patient-derived cells are those with the most pronounced glycolytic phenotype, whereas A2780-resistant cells mainly diverge from the others due to alterations in a few specific metabolites already known as markers of resistance.

## 1. Introduction

Ovarian cancer (OC) is the fifth most common cause of cancer death in women and the most lethal of all gynecological diseases, with 46% 5-years survival after diagnosis. High-grade serous epithelial ovarian cancer (HGSOC) is the most frequent histotype [1]. Usually, patients with OC have locally advanced disease at diagnosis, with diffuse carcinosis in the lower, medium, and upper abdomen. The cornerstone of treatment for advanced OC has long been a combination of maximal effort cytoreductive surgery and chemotherapy. The combination of carboplatin and paclitaxel has been long established as the first line of treatment in OC, either in the neoadjuvant, adjuvant, or palliative setting [2]. However, many patients with advanced disease will subsequently relapse despite treatment, with the time to relapse being a negative prognostic indicator for survival and for response to further platinum-based treatment [3]. Indeed, despite the initial high sensitivity to chemotherapy and complete clinical response, chemotherapy resistance is a common occurrence at relapses, and thus, there is a significant unmet demand for improved treatments [1,3].

Although cell lines represent a valid and consolidated tool for the study of several biological and pathological processes, they present some drawbacks that are worth taking into account. Several studies have shown that cell lines overexpress genes involved in drug resistance; additionally, the transcriptome profiles of immortalized cells deriving from different tumor types bear more resemblance to each other than to the clinical samples they are supposed to represent [4,5]. Concerning OC, there are merely 100 cell lines described in the literature, and many of them are largely used to investigate the characteristics of this complex and multifaceted cancer. Among them, the A2780 cell line is one of the most used cancer cell lines. In fact, the search for “A2780” in PubMed resultes in more than 3900 studies to date. The A2780 cell line is derived from an ovarian endometrioid adenocarcinoma of an untreated patient. Its cisplatin-resistant counterpart has been established in several laboratories and can also be found in ECACC cell lines collection (A2780cis); thus, A2780 is among the few models of isogenic platinum-resistant OV cell lines (importantly, none of the existing is assigned as high grade serous) [6].

As cancer causes significant metabolic alterations to sustain its growth, metabolomics mass spectrometry and nuclear magnetic resonance (NMR) approaches have been widely applied to learn about the biochemistry of cancer cells [7,8]. Several metabolomic studies have been performed on OC, both using cancerous tissues and selected cell lines, in order to better characterize the differences between normal and cancerous tissues/cells and cisplatin-sensitive and resistant cancer cell lines [9,10,11,12]. These studies have pointed to alterations of different metabolomic patterns in response to various toxic drugs and/or in comparison between platinum-sensitive and platinum-resistant counterparts [13,14,15,16,17,18,19,20].

As an example, some of us characterized the cellular effects induced in A2780 cells and A2780cis cells by treatment with the three clinically established platinum drugs, i.e., cisplatin, carboplatin, and oxaliplatin [21]. With the same methodological approach, we also addressed the cellular alterations caused by gold(I)-based drugs with optimal anticancer properties [22,23,24].

Being A2780 among the most popular cell line models for ovarian cancer, here we would like to highlight metabolomic similarities and differences with primary cells of HGSOC, which, indeed, represent the most common histological type of ovarian cancer.

To this aim, we analyzed and compared via ^1^H NMR the intracellular and extracellular metabolome of HGSOC cells derived from (i) macroscopically cancerous solid tissue (HGSOC_T) and (ii) ascites (HGSOC_A) collected from HGSOC patients at the time of cytoreductive surgery with those of A2780 and A2780 cisplatin-resistant (A2780cis) cell lines.

To the best of our knowledge, no previous studies investigated the NMR-based untargeted metabolomic profile of primary OC cells in comparison to established cell lines. We found a striking similarity in the metabolomic profile of primary cells derived from different donors. However, these cells present interesting differences with respect to both A2780 and A2780cis cells, which are discussed extensively.

## 2. Materials and Methods

### 2.1. Tissue Collection and High-Grade Serous Ovarian Cancer Cell Isolation

Ovarian cancer samples and ascites were obtained from a total of 10 patients (5 for solid tumors and 5 for ascites) at the time of primary cytoreductive surgery for advanced HGSOC. The patients were enrolled at the Obstetrics and Gynecology Unit, Careggi University Hospital of Florence. Enrolled patients signed the written informed consent approved by the Tuscany Region Ethics Committee (protocol number 14780). Tissue samples were collected from areas of clearly macroscopic cancerous tissue. Primary cells from ascites and solid tumors are derived from different patients. Ascites and cancerous tissues were immediately transferred to the laboratory. Unless otherwise specified, all cell culture products were purchased from Euroclone S.p.A. 20016 Pero (MI), Italy. Solid samples were maintained in sterile phosphate-buffered saline (PBS) and processed for cell isolation within 30 min of collection. HGSOC cell isolation from solid tissues (HGSOC_T) and ascites (HGSOC_A) was performed according to the protocol of TG Shepherd et al. [25], with minor modifications. In particular, tissues were digested with Dispase II, 2 mg/mL (Sigma, Milan, Italy in RPMI 1640 medium for 30 min and then cultured in a medium composed of 10% fetal calf serum (FCS) and an equal amount of RPMI 1640 and M199 medium supplemented with glutamine and antibiotics in order to minimize bacterial contamination. The medium was changed after 48 h in order to allow ovarian cancer cells to adhesion. For cell isolation from ascites, ascitic fluid was mixed 1:1 with the complete RPMI 1640 and M199 medium. After adhesion, the cells were maintained in RPMI and M199 complete medium. Confluent cells were split 1:2 and always used before passage five.

### 2.2. Characterization of HGSOC Cells

Cells were examined under reverse-phase optical microscopy to verify epithelial morphology. Western blot against Ep-CAM (GTX113091 Gene Tex, Irvine, CA, USA) and CA125 (ORB256698 Biorbyt, Durham, NC, USA) were performed to confirm the epithelial origin and tumor phenotype of the isolated cells.

### 2.3. Cell Lines

A2780 and A2780is cells were purchased from Creative Bioarray, Frankfurt, Germany, (A2780: human ovarian carcinoma; catalogue no.: CSC-C9491J, passage no.: P + 8. A2780cis: cisplatin-resistant human ovarian carcinoma; catalogue no.: CSC-C9492J, passage no.: P + 8). Cell cultures were grown in the same medium used for primary ovarian cancer cells. Cells were sub-cultured twice weekly, splitting them 1:5 (3–6 × 10^4^ cells per cm^2^). All reagents were purchased from Euroclone.

### 2.4. IC_50_ Determination

The inhibition of cell proliferation by cisplatin was evaluated through MTT (3-(4,5-dimethylthiazol-2-yl)-2,5-diphenyltetrazolium bromide) test. Cells were seeded in 96 well microplates at a density of 10,000 cells so that the well could have exponentially growing cells. After 24 h, cells were treated with cis-Pt at concentrations ranging from 0.1 to 100 µM and incubated for 72 h. For the test, cells were incubated with 0.5 mg/mL MTT for 1 h at 37 °C. Blue formazan formed after MTT precipitation was dissolved in DMSO, and optical density at 595 nm was evaluated in a microplate reader interfaced with Microplate Manager/PV version 4.0 software (BioRad, Hercules, CA, USA). GraphPad Prism software version 6.0 (Graphpad, Boston, MA, USA) was used to calculate the half-maximal inhibitory concentration (IC_50_).

### 2.5. NMR Sample Preparation and NMR Analysis

Cell lysates and the respective growth media were analyzed using an untargeted ^1^H NMR-based metabolomics [26]. For A2780 and A2780cis cells, the NMR experiments were performed on biological triplicates (i.e., from different culture batches). For HGSOC cells, the NMR experiments were performed on 10 samples of ovarian cancer cells, each isolated from a different patient with advanced HGSOC (5 from solid tissues, HGSOC_T, and 5 from ascites, HGSOC_A).

HGSOC cells and A2780 cell lines were grown in the same medium (10% FCS and an equal amount of medium RPMI and medium M199 supplemented with glutamine and antibiotics) until a confluence of 70–80%. A sample for NMR was prepared by first washing the cells with PBS and then scraping them in a solution of PBS containing a protease and phosphatase inhibitor cocktail (Sigma, Milan, Italy).

The inhibitor cocktail was diluted in DMSO, and it was used to quench enzymatic reactions and stabilize the cellular metabolome; the amount of DMSO present in the buffer lysis does not affect the sample metabolome in any way. Cells were lysed by sonication in ice and then centrifuged at 200,000× *g* for 30 min at 4 °C. For the exo-metabolome analysis, 1 mL of the growth medium was centrifuged in order to eliminate non-attached cells. All the samples were stored at −80 °C until analyses.

A Bruker 600 MHz spectrometer (Bruker BioSpin, Rheinstetten, Germany), which operates at 600.13 MHz proton Larmor frequency and is optimized for metabolomic investigations, was used to record all of the ^1^H NMR spectra. The spectrometer is furnished with a 5 mm PATXI ^1^H–^13^C–^15^N and ^2^H-decoupling probe, which includes a z-axis gradient coil, an automatic tuning-matching, and a BTO 2000 thermocouple for temperature stabilization.

Samples were held inside the NMR probe head for five minutes before the measurements in order to equilibrate the temperature at 300 K.

In the case of cell lysates- to avoid sample degradation during the analysis-frozen samples were thawed and analyzed one at a time. The NMR samples were prepared by adding 50 µL of ^2^H_2_O to 450 µL of each lysate sample. After being vortexed to homogenize the mixtures, they were put into Bruker BioSpin srl 5 mm NMR tubes for examination. Cell lysate spectra were obtained using the Carr–Purcell–Meiboom–Gill (CPMG) sequence using a one-dimensional (1D) spin-echo sequence with water presaturation; the following acquisition parameters were used: 512 scans, 73,728 data points, a spectral width of 12,019 Hz and a relaxation delay of 4 s.

The CPMG pulse sequence is designed to suppress signals originating from macromolecules present in the sample, such as lipids and proteins, and to allow for the selective detection of low-molecular weight molecules such as metabolites. The stability of the metabolomic profile of the samples following these procedures was carefully checked on the time scale of the NMR sample acquisition (about 90 min per sample), Figure S1.

In the case of cell culture media, all the samples were thawed and analyzed together using an automatic refrigerated sample changer (SampleJet, Bruker BioSpin, Rheinstetten, Germany). For the sample preparation, 300 µL of each medium sample was mixed with 300 µL of a sodium phosphate buffer (70 mM Na_2_HPO_4_; 20% *v*/*v* ^2^H_2_O; 4.6 mM TMSP, pH 7.4), homogenized by vortexing and transferred into 5 mm NMR tubes (Bruker BioSpin srl, Rheinstetten, Germany). The NMR growth media spectra were obtained using the 1D nuclear Overhauser enhancement spectroscopy (NOESY)-presaturation pulse sequence; the following acquisition parameters were used: 64 scans, 98,304 data points, a spectral width of 18,028 Hz, and a relaxation delay of 4 s.

The 1d NOESYpulse sequence allows the simultaneous detection of the signals from metabolites and macromolecules; however, in the cell growth media, the latter contribution to the NMR spectra is negligible.

Prior to applying the Fourier transform, the raw data were multiplied by an exponential line broadening at 0.3 Hz. Using TopSpin 3.6 (Bruker Biospin srl, Rheinstetten, Germany), transformed spectra were automatically corrected for baseline and phase distortions.

### 2.6. Statistical Analysis

The statistical analyses reported in this study were performed using the “R” software (https://www.r-project.org/). Multivariate statistics were applied to spectral buckets. To obtain the spectral buckets, each spectrum was divided into 0.02 ppm chemical shift bins from 10.00 ppm to 0.2 ppm and integrated using the AMIX 4.0.2 software. Each bin was normalized to the total spectral area, calculated without the water and DMSO regions (4.50–5.00 ppm and 2.90–2.60 ppm, respectively).

To get a broad picture of the data and find clusters, an unsupervised exploratory technique, i.e., the Principal Component Analysis (PCA), was used. Leave-one-out cross-validation was used to validate the PCA models, considering the number of components that explain 99% of the variance of the dataset.

Using an internal R script, the metabolites with well-resolved peaks in their spectra were identified, and their concentrations were examined. We identified and quantified 33 metabolites in the cell lysate spectra and 28 in the growth medium spectra. The metabolite assignment was performed using the BBIOREFCODE (Bruker BioSpin, Rheinstetten, Germany) ^1^H NMR spectral library of pure compounds. The ambiguities were solved using spiking experiments. Matching between new NMR data and databases was performed using the AMIX 4.0.2 software (Bruker BioSpin, Rheinstetten, Germany).

The altered metabolites among the different groups were identified using the non-parametric Wilcoxon–Mann–Whitney test; False Discovery Rate correction (FDR) was used- with the Benjamini and Hochberg method- to minimize false discoveries; a *p*-value < 0.05 was deemed statistically significant.

For each metabolite, the Log_2_ Fold change (FC) was computed to show the variation in metabolite levels between A2780cis or HGSOC cells compared to A2780 cells. FC was calculated as the ratio of the median metabolite concentrations in the spectra of HGSOC or A2780cis over A2780. The metabolites of the growth media were grouped into two types: those that are released into the medium during cellular growth and those that are taken up from it. For the metabolites that are taken up, lower concentration levels result in greater consumption of nutrients or increased uptake; for the metabolites that are released, indeed, lower concentration levels result in a lower release of the molecules (and vice versa).

## 3. Results and Discussion

**Figure 1:**
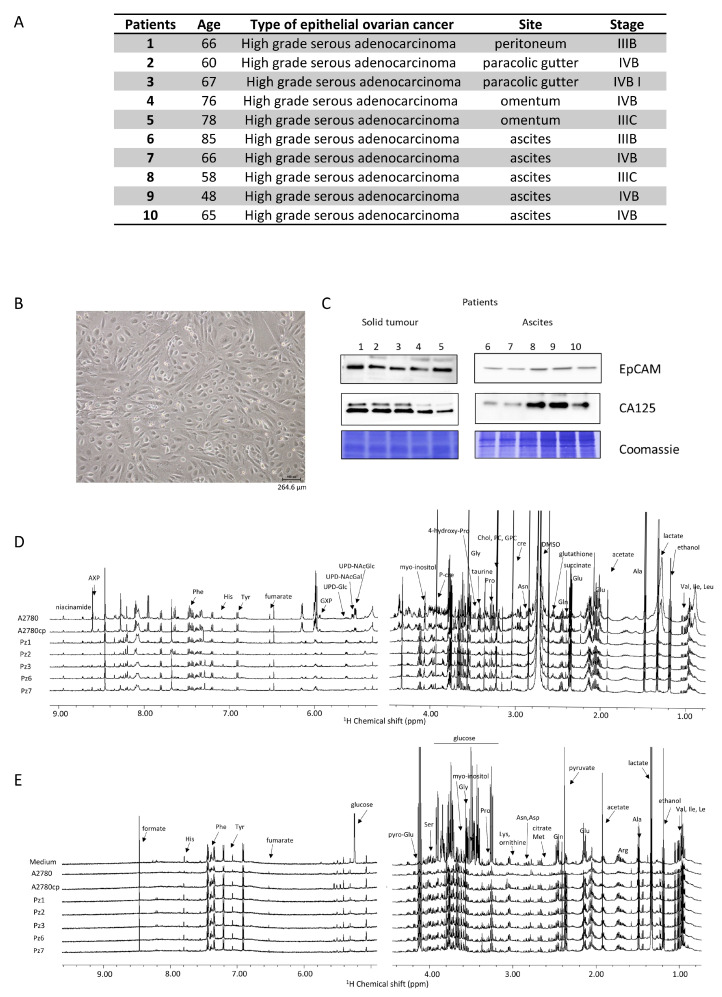
Characterization of HGSOC cells. (**A**) main features of the donors; (**B**) bright field image of representative HGSOC cells (scale bar = 264.6 µm) (**C**) western blot analysis of protein extracts of HGSOC cells showing the expression of EpCAM and CA125 proteins as measured using specific antibodies. Representative ^1^H NMR CPMG spectra of (**D**) cell lysates (endo-metabolome) and (**E**) growth media (exo-metabolome) of HGSOC_T cells and HSGOC_A in comparison with A2780 and A2780cis (left: downfield, 5.00–9.00 ppm; right: upfield, 1.00–4.50 ppm). Spectra were recorded at 600 MHz and 300 K. All metabolites that have been identified in the spectra are indicated.

The main features of the HGSOC donors are reported in Figure 1A. All the patients presented a diagnosis of high-grade serous ovarian cancer, and samples were collected during primary cytoreduction surgery and before chemotherapy. All the isolated HGSOC cells had an epithelial morphology, as shown in Figure 1B for a representative case. In order to confirm the epithelial origin, a western blot analysis against the EpCAM (epithelial cell adhesion molecule) glycoprotein was performed [27,28]. Furthermore, since 90% of OC express the cancer antigen 125 (CA125) [29], its expression was also determined [27,28]. As shown in Figure 1C, the HGSOC cells isolated from the ten donors all express both EpCAM and CA125 proteins.

**Figure 2:**
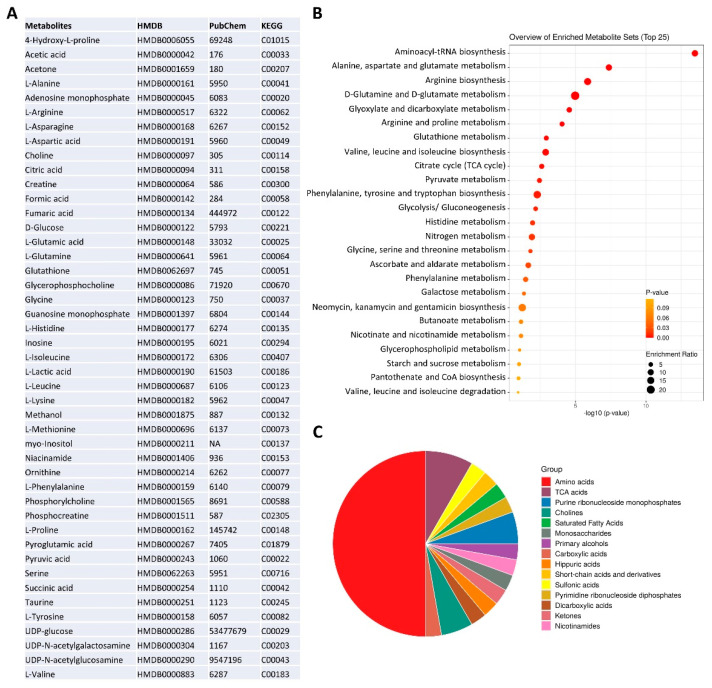
NMR metabolomics phenotype of the cells. (**A**) metabolite identification with KEGG and HMDB codes. Enrichment analysis: (**B**) pathway-based on KEGG and (**C**) chemical structures, using MetaboAnalyst 6.0 software [30].

The NMR metabolomic fingerprints of the 10 different HGSOC primary cell cultures (five isolated from solid tumors, HGSOC_T, and five isolated from ascites, HGSOC_A) were analyzed and compared to those of the immortalized A2780 ovarian cancer cells and of the A2780cis cells. The metabolomic fingerprints of the different cell lines were characterized by acquiring ^1^H NMR CPMG spectra of their cell lysates (endo-metabolome, Figure 1D) and ^1^H NMR NOESY spectra of their growth media (exo-metabolome, Figure 1E); samples of lysates and media were analyzed separately. In the endo- and exo-metabolome, we were able to assign 33 and 28 molecules, respectively; these molecules are the most abundant metabolites (concentration > 1–10 μM) and take part in the main metabolic pathways (Figure 2), thus providing an overall picture of the metabolic phenotype of the cells.

Notably, in the three cell types (i.e., A2780, A2780cis, and HGSOC cells), we observed differences in the relative concentrations of the metabolites but not in their chemical nature. Exceptions are the signals of a few molecules, i.e., two UDP-sugars (N-acetyl-glucosamine and N-acetyl-galactosamine, UDP-NAcGln and UDP-NAcGal), which are visible only in the lysates of the immortalized cell lines, and the signals of UDP-glucose (UDP-Glu) and inositol, which are only present in the lysate of HGSOC cells (Figure 1D). Our NMR metabolomic approach was not able to discriminate among the different phosphorylation statuses of adenine and guanine nucleotides, so we generally refer to AXP and GXP in the rest of the manuscript. In the spectra of the growth media, no differences in the nature of secreted metabolites are observed.

**Figure 3:**
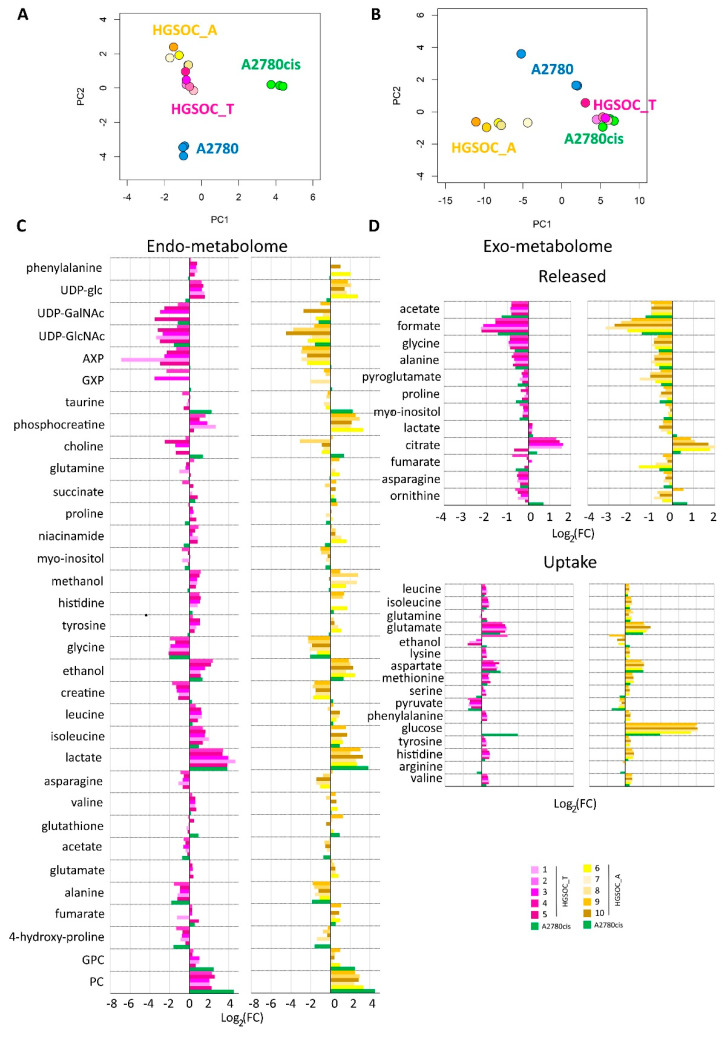
Score plots of the PCA analyses performed on the bucketed NMR spectra of (**A**) cell lysates PC1 (57.4%) vs. PC2 (28.0%) and (**B**) growth media, PC1 (89.1%) vs. PC2 (7.6%). Each dot represents a different NMR sample, with the following color coding: A2780 (blue), A2780cis (green), HGSOC_T (magenta), and HGSOC_A (yellow). Bar plots (**C**,**D**) are used to compare the metabolite concentration in HGSOC_T or HGSOC_A and A2780cis cells with respect to the A2780 cells. Positive/negative bars indicate higher/lower concentrations in HGSOC and A2780cis cells with respect to A2780 cells (Log_2_(FC)).

Multivariate unsupervised PCA analyses were performed on the ^1^H NMR CPMG spectra for cell lysates and on the ^1^H NOESY spectra for the growth media separately. Each of these PCAs clearly shows a strong remodeling of the endo- and the exo-metabolomic phenotype of A2780 cells upon the development of the cisplatin resistance. In fact, A2780 cells and their resistance counterpart A2780cis cells cluster separately in the PCA score plots, with a 100% discrimination accuracy between the two groups (Figure 3).

When considering the endo-metabolome (Figure 3A), both HGSOC_T and HGSOC_A cells cluster about halfway between A2780 and A2780cis cells, and the phenotype of all the primary cultures appears very similar. Instead, when considering the exo-metabolome HGSOC_T cluster very close to the A2780cis cells while HGSOC_A cells cluster separately from both A2780, A2780cis and HGSOC_T cells (Figure 3B); from these data it results a striking similarity in the metabolomic profile of primary cells derived from different donors, both for HGSOC_T group and for HGSOC_A group. Moreover, it results that from the endo-metabolome point of view, the primary cells, independently from their origin, share some common characteristics with the sensible A2780 and others with the A2780cis cells. Contrarily, from the exo-metabolome point of view, the behavior of HGSOC_T cells (but not of HGSOC_A) is more similar to that of A2780cis cells than to that of A2780 cells. Notably, a closer similarity between HGSOC (independently from the origin) and A2780cis cells is also observed in terms of the changing 50% inhibitory concentration (IC_50_) of cisplatin determined via the MTT assay to evaluate cell viability: the IC_50_ at 72 h value for HGSOC falls in the 7–11 μM range (11.5 ± 3.1 for HGSOC_T and 7.6 ± 2.1 for HGSOC_A), which is comparable with the corresponding values of 8.6 ± 0.9 μM of A2780cis; the IC_50_ value for A2780 cells is of the order of 2.7 ± 0.66 μM (Figure S2).

Figure 3C,D clearly show the differences in the relative amount of the intracellular and extracellular metabolites in HGSOC_T, HGSOC_A, and A2780cis cells with respect to A2780 cells.

**Figure 4:**
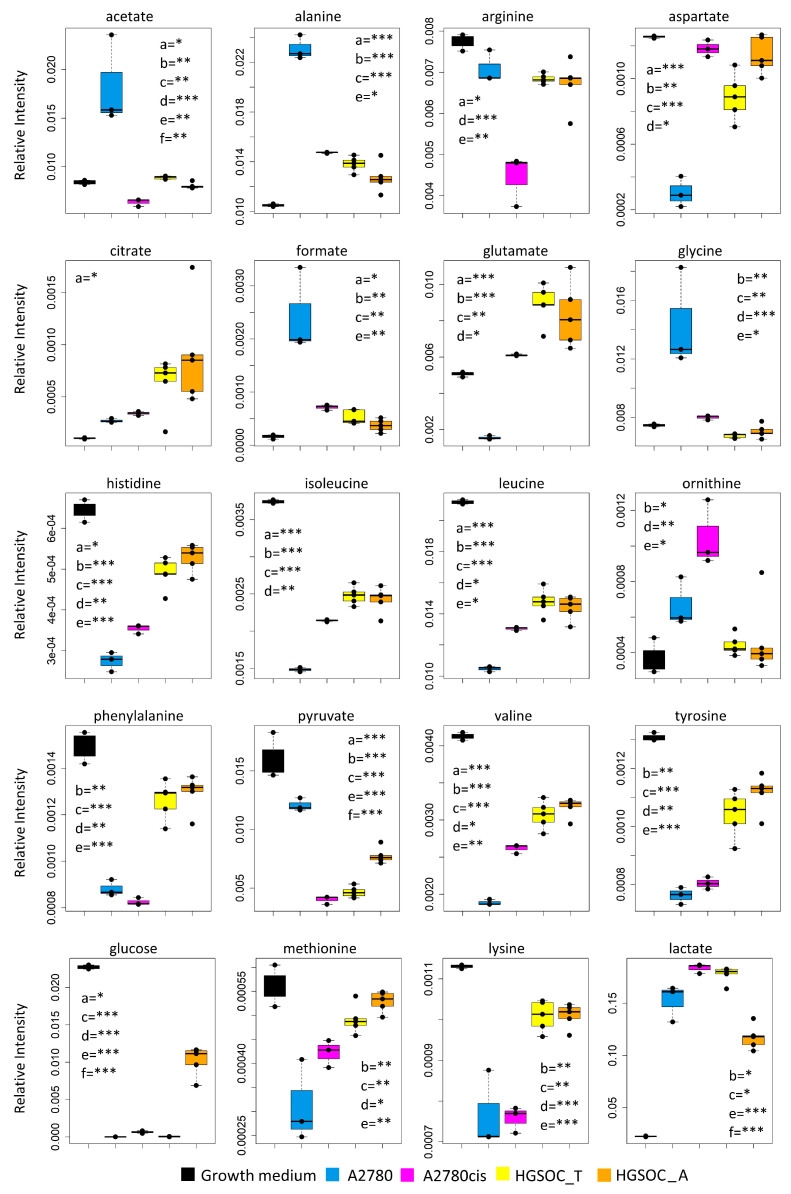
Box plots showing the relative concentration of extracellular metabolites that result significantly altered in the different cell lines. Color coding: A2780 (blue), A2780cis (magenta), HGSOC_T (yellow), HGSOC_A (orange), growth medium(black). The letters a–f indicate the different comparisons, i.e., a = A2780 vs. A2780cis; b = A2780 vs. HGSOC_T; c = A2780 vs. HGSOC_A; d = A2780cis vs. HGSOC_T; e = A2780cis vs. HGSOC_A; f = HGSOC_T vs. HGSOC_A. * indicates 0.05 < *p*-value < 0.01; ** indicates 0.01 < *p*-value < 0.001; *** indicates *p*-value > 0.001.

**Figure 5:**
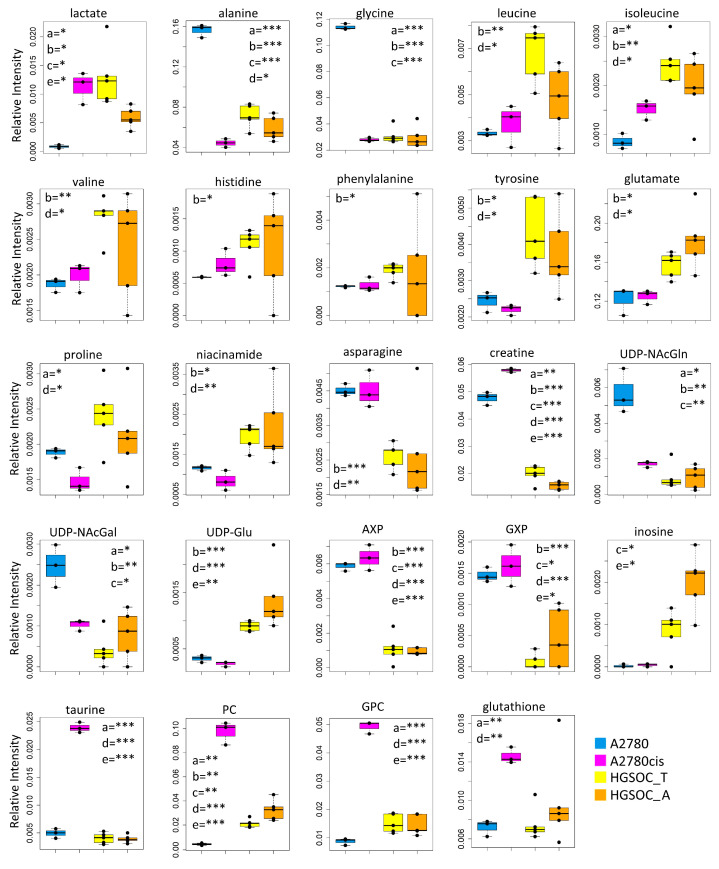
Box plots showing the relative concentration of intracellular metabolites that result significantly altered in the different cell lines. Color coding: A2780 (blue), A2780cis (magneta), HGSOC_T (yellow), HGSOC_A (orange). The letters a–e indicate the different comparisons, i.e., a = A2780 vs. A2780cis; b = A2780 vs. HGSOC_T; c = A2780 vs. HGSOC_A; d = A2780cis vs. HGSOC_T; e = A2780cis vs. HGSOC_A. * indicates 0.05 < *p*-value < 0.01; ** indicates 0.01 < *p*-value < 0.001; *** indicates *p*-value > 0.001.

Concerning the metabolism of individual amino acids, the HGSOC_T, HGSOC_A, and A2780cis cells show a strong reduction of the consumption of Asp and Glu from the media, and also the uptake of the branched-chain amino acids (Val, Ile, Leu) and of the aromatic amino acids (His, Phe and Tyr) is significantly reduced (Figure 3D and Figure 4) in comparison to A2780. On the other hand, both HGSOC_T, HGSOC_A, and A2780cis cells show decreased release of Ala and Gly. Considering intracellular amino acids, Ala and Gly levels are much lower in A2780cis, HGSOC_T, and HGSOC_A, whilst the intracellular levels of BCAA and aromatic amino acids increase regularly in the order A2780 < A2780cis < HGSOC A and HGSOC_T (Figure 3D, Figure 4 and Figure 5). These data suggest that in primary cell cultures and in A2780cis, there is an increased consumption of Ala and Gly, while branched-chain amino acids together with Glu and Asp seem to be less used.

Although the role of glycine in cancer is debated and could be cell-type specific [31], it is interesting to underline that Jain M et al., measuring the consumption and release of 219 metabolites from media across 60 cancer cell lines, found a correlation between glycine consumption and rapidly proliferating ovarian, colon and melanoma cells [32]. The rapidly growing cancer cells, like HGSOC, appeared to need glycine for the synthesis of purine nucleotides required for the continued synthesis of DNA. In line with this hypothesis, both A2780cis and primary ovarian cancer cells show a decreased excretion of format, another metabolite involved in purine nucleotide synthesis.

Concerning the energetic metabolism, in A2780 and HGSOC_T cells at the time of cell harvesting, glucose was completely consumed in the media of the cells. Very low glucose levels are instead visible in A2780cis spectra. HGSOC_T and A2780cis cells show a higher uptake of pyruvate from the media with respect to A2780 cells (log_2_(FC) = 1.36 for HGSOC_T, and log_2_(FC) = −1.48 for A2780cis) (Figure 3D and Figure 4). Pyruvate represents a central node in metabolism: it can be converted in acetyl-coA by the pyruvate dehydrogenase complex and thus directed into the TCA cycle, or it can be used to synthesize alanine and glycine and reduced to lactate in a lactic fermentation. The extracellular amount of lactate was not significantly different among A2780, A2780cis, and HSGOC_T, even if slightly higher in HGSOC_T and A2780cis cells, whereas the intracellular concentration of lactate is higher in HGSOC_T (log_2_(FC) = 3.84), and A2780cis (log_2_(FC) = 3.81) cells then in A2780 cells (Figure 3C,D and Figure 5). Furthermore, a relevant increase in excreted citrate is observed for HGSOC_T (even if its variation is not significant after FDR correction); also, for A28780cis, a slightly higher excretion of citrate is observed (Figure 3D and Figure 4). Since citrate is an allosteric inhibitor of phosphofructokinase 1, the key regulatory enzyme of glycolysis, maintenance of its low intracellular level ensures a high flow of glycolysis [33,34]. Altogether, the described behavior points to a glycolytic phenotype, which increases in the order A2780 < A2780cis < HGSOC_T.

Although the metabolic behavior of A270cis and HSGOC_T is very clear with respect to those of A2780 cells, the HGSOC_A showed not easily explainable trends in the levels of the energetic metabolites. In particular, HGSOC_A showed very high levels of extracellular glucose along with lower levels of lactate excretion with respect to all the other cell types. These trends may reflect lower glycolytic flux, but the extracellular concentrations of pyruvate and citrate are very similar to the levels in HSGOC_T cells (Figure 3D and Figure 4). It is important to note that the high glucose- and low lactate- levels in the NMR spectra of the growth media of the HGSOC_A group are the variables responsible for the clusterization of this group in the PCA score plot in a different area from HGSOC_T and A2780cis groups.

The only significant feature that discriminates A2780cis cells from HGSOC cells at the level of the exo-metabolome concerns metabolites involved in the urea cycle. Increased ornithine release along with increased Arg uptake characterize A27080cis cells but have no counterpart in HGSOC cells (Figure 3D and Figure 4). These changes are evocative of a dysregulation of the urea cycle featuring A2780cis-resistant cells.

At the level of the endo-metabolome, there are several features that cause HGSOC_T and HGSOC_A cells to occupy a characteristic position in the “metabolic space”, which is intermediate between those of A2780 and A2780cis cells (see PCA score plot in Figure 3A). The concept is illustrated by the box plots in Figure 5.

In the A2780cis cell line, we observed decreased intracellular levels of UDP-NAcGlc and UDP-NAcGal. In both HGSOC cell types, the signals of these two metabolites are below the detection limit. On the contrary, the signals of UDP-Glu are well visible in HGSOC_T and HGSOC_A spectra and below the detection limit in both the A2780 and A2780cis cells (Figure 3C and Figure 5). UDP-sugars and, in particular, UDP-NAcGlc are involved in the hexosamine biosynthetic pathway (HBP). High glucose levels in the cytoplasm of cancer cells not only contribute to increased glycolysis but also increase carbon flux into side-branch pathways, including HBP [35]. HBP converts glucose in UDP-NAcGlc, a high-energy donor for protein glycosylation, and there is evidence that altered glycosylation in cancer affects cell signaling pathways [36]. Interestingly, the trend of decrease in UDP-NAcGlc and UDP-NAcGal concentrations is opposite with respect to the degree of glycolytic phenotype of the cells under study, suggesting that the consumption of these metabolites is higher in more glycolytic cells. In line with this, the decrement of UDP-NAcGlc and NAcGal are considered markers for cancer aggressiveness [21,37].

In the endo-metabolome, the concentrations of AXP and GXP are also much lower in HGSOC_T and HGSOC_A cells with respect to the other two lines (Figure 3C and Figure 5); in particular, the quantity of GXP is essentially negligible. On the contrary, inosine that is under the detection limit in A2780 and A2780cis cells is well detectable in HGSOC_T and HGSOC_A cells. This observation is difficult to rationalize; it has been reported that inosine released by dead and dying cells mediates tumor cell proliferation via purinergic receptors [38], but here, we could only observe increased intracellular levels and not release into the media.

When it comes to the intracellular levels of niacinamide, glutamate, proline, asparagine, and creatine, the HGSOC cells are quite different from both sensitive and resistant A2780 cells, which instead are relatively similar to one another; the first three metabolites result in significantly increased in HGSOC cells, while the other two are significantly decreased (Figure 5).

A2780cis cells show very high levels of phosphocholine (PC, log_2_(FC) = 4.49) and glycerophosphocholine (GPC, log_2_(FC) = 2.44) with respect to A2780 cells. The same trend is also present in HGSOC_T and HGSOC_A, although with a markedly less pronounced trend (average log_2_(FC) = 2.25 and 2.86 for PC and 0.63 and 0.43 for GPC, in HGSOC_T and HGSOC_A, respectively) (Figure 3C and Figure 5). Consistently, literature data reports that cisplatin-resistant and responsive A2780 cells have different phosphocholines profiles [39], which can be explained in terms of biophysical changes in the membranes of resistant cells, which in turn might influence drug transport. Furthermore, PC changes in the progression from non-tumoral ovarian surface epithelial cells versus carcinoma cells and dysregulation of choline metabolism have been proposed as hallmarks of novel cancer [40].

The intracellular levels of taurine and, to a lesser extent, glutathione are also greatly increased in A2780cis with respect to A2780, HGSOC_T, and HGSOC_A cells, which instead are very similar to each other (Figure 3C and Figure 5). These features seem, therefore, associated specifically with the resistance developed following treatment with cisplatin. It is known that A2780 cell line cisplatin resistance correlates with an increased intracellular accumulation of glutathione and taurine that, in turn, protects cells from oxidative stress [41], volume reduction [42], and apoptosis [43]. Furthermore, the TauT transporter, involved in taurine transport across the membrane, promotes survival and multidrug resistance in colorectal cancer cells [44], and its overexpression protects kidney cells against cisplatin-induced cell death [45]. Thus, in an A2780cis cell line, the increased level of intracellular glutathione and taurine could be the main mechanisms involved in cisplatin resistance; on the other hand, HGSOC_T and HGSOC_A cells have a glutathione and taurine content similar to the A2780 sensitive cell line. Thus, the intrinsic less sensitivity to cisplatin of these cells (as demonstrated by the high value of 72 h IC_50_) could be dependent on other factors.

It should also be considered that the primary cells used in this study are from no-treated patients, whilst the A2780cis cell line is obtained after a long period of treatment with a sub-lethal concentration of the drug; thus, it is possible that the increased glutathione and taurine level is a consequence of the treatment itself. In fact, some of us have recently demonstrated that the treatment of the A2780 cell line with platinum compounds for 48h leads to an increase of intercellular levels of taurine and glutathione (even if the latter is not significant) [21].

## 4. Conclusions

This pilot study shows how NMR-based metabolomics can reveal differences among different cell lines. Tumour tissues and ascites from 10 volunteers were processed to establish cells under optimized culture conditions. In spite of the finite lifespan of primary cells and their limited replicative capacity [46], we could successfully obtain enough samples for metabolomics profiling via ^1^H NMR.

The metabolome of the isolated HGSOC cells was compared to those of A2780 cells and its parent cisplatin-resistant A2780cis line. A2780 cells are commonly used as a model for OV to study the potency of chemicals, methods of delivery, and treatments. The exact histological origin of A2780 is not specified in the original reference, and it is indicated as “tumor tissue” [47]. On the basis of the genetic profile, A2780 cells do not closely resemble HGSOC [47], but, as recently described by HAllas Potts A. et al. [48], ovarian cancer cell lines do not all behave as expected based on their putative identity. For example, concerning migration, A2780 behaved similarly to PEO1, PEO4, and FUOV1, which are HGSOC cell lines; COV318 cells, instead, are genomically classified as a high-grade serous cell line but behave similarly to non-serous cell lines.

Our data suggests very high similarity in the metabolomic profile of primary cells derived from different patients, both for the HGSOC_T group and for the HGSOC_A group. Their exo-metabolic and partially endo-metabolomic profiles are very similar to those of A2780cis cells, which, in turn, result in a more realistic model in comparison to A2780. As discussed above, the main differences at the levels of the endo-metabolome could be restricted to the metabolites related to the mechanisms of acquired resistance upon drug treatment. Analyzing metabolic profiles from ex vivo primary cell cultures post six cycles of platinum-based chemotherapy would be of high interest. However, obtaining such samples is challenging due to the rarity of secondary cytoreductive surgery in platinum-refractory patients. The scarcity of these samples could pose a significant limitation to conducting such studies [49,50].

Based on these results, one could envisage organizing large-scale studies where the metabolome fingerprint of each patient is used to identify predictive biomarkers of the individual response to cisplatin treatment (including the development of resistance). This type of approach would require following the patients on a time scale of years but, if successful, could be suitable for translation into the clinical practice.

Another limit of the study regards the use of RPMI medium for the experiments; although classical culture media such as RPMI have been extensively used in studies involving A2780 cells as well as other ovarian cancer cell models, recent studies have shown that the non-physiological concentration of some metabolites in their compositions impacts on cell physiology [51,52,53]. For example, in classical DMEM composition, glucose and pyruvate concentrations much exceed their normal concentrations in plasma. A high level of glucose can induce oxidative stress and reduce oxidative phosphorylation [54,55], while a high level of pyruvate can reduce the use of glutamine as a carbon source for the Krebs cycle [56]. To solve these problems, recently, growth media with a composition more similar to that of human plasma were formulated, and their use has been suggested in studies aimed at characterizing cell metabolism. In particular, Human Plasma-LikeMedium [57] and Plasmax [53] are the most used.

To our knowledge, human-like media have never been employed in metabolomic studies on these cell types, and there is no evidence that primary ovarian cancer cells can be established in these media. For these reasons, we performed the present study in a standard RPMI medium. It is also worth noting that RPMI contains a low level of glucose and no pyruvate. Thus, although we cannot exclude the non-physiological effects of other metabolites, the important concerns regarding the effect of high levels of glucose and pyruvate can be limited or even excluded.

In the future it will certainly be interesting to explore the possibility of establishing primary ovarian cancer cells in human-like media; the use of these media both for primary and established ovarian cancer cells could open up scenarios partly masked in current culture media. Moreover, our method can be used to test other OV cell lines, in order to find the most appropriate in vitro model for metabolic studies of HGS tumours.

## Data Availability

This study is available at the NIH Common Fund’s National Metabolomics Data Repository (NMDR) website, the Metabolomics Workbench [58], https://www.metabolomicsworkbench.org (accessed on 3 October 2022) where it has been assigned Study ID ST002116. The data can be accessed directly via its Project DOI: http://dx.doi.org/10.21228/M8CM41 (accessed on 3 October 2022).

## Supplementary Materials

The following supporting information can be downloaded at: https://www.mdpi.com/article/10.3390/cells13080661/s1, Figure S1: Stability test. Relative concentration levels of metabolites in a cell lysate sample, prepare with different concentrations of protease and phosphate inhibitor cocktail (0.5× and 1×) and acquired at different time points. Blue crosses: T0, orange crosses: T = 30 min, gray crosses: T = 60 min, yellow crosses: T = 90 min and cyan crosses: T = 120 min; Figure S2: (A) Representative cytotoxicity-curves of cisplatin after 72 h of treatments determined by MTT assay. (B) Histogram reports media ± SD of IC50 measured in HGSOC_T, HGSOC_A, A2780 and A2780cis cells. Statistical analysis was performed with Anova using GraphPad Prism 6.01 software; * indicates *p*-value < 0.05. ** indicates *p*-value < 0.01.
