# Supplementary material for: NMR Metabolomics of Primary Ovarian Cancer Cells in Comparison to Established Cisplatin-Resistant and -Sensitive Cell Lines
Source: Cells. 2024 Apr 9;13(8):661. doi: 10.3390/cells13080661 (PMC11049548; doi:10.3390/cells13080661)
Supplement: Supplementary file 1 [file cells-13-00661-s001.zip › cells-2920163-supplementary.pdf]

NMR metabolomics of primary ovarian cancer cells in comparison to established cisplatin-resistant and sensitive cell lines.

Veronica Ghini[1,2*], Flavia Sorbi[3], Massimiliano Fambrini[3], Francesca Magherini[3*]

[1]Department of Chemistry, University of Florence, Italy.

[2]Magnetic Resonance Center (CERM), University of Florence, Italy.

[3]Department of Experimental and Clinical Biomedical Sciences "Mario Serio" University of Florence Viale G.B. Morgagni 50, 50134, Florence, Italy,

Supporting Information

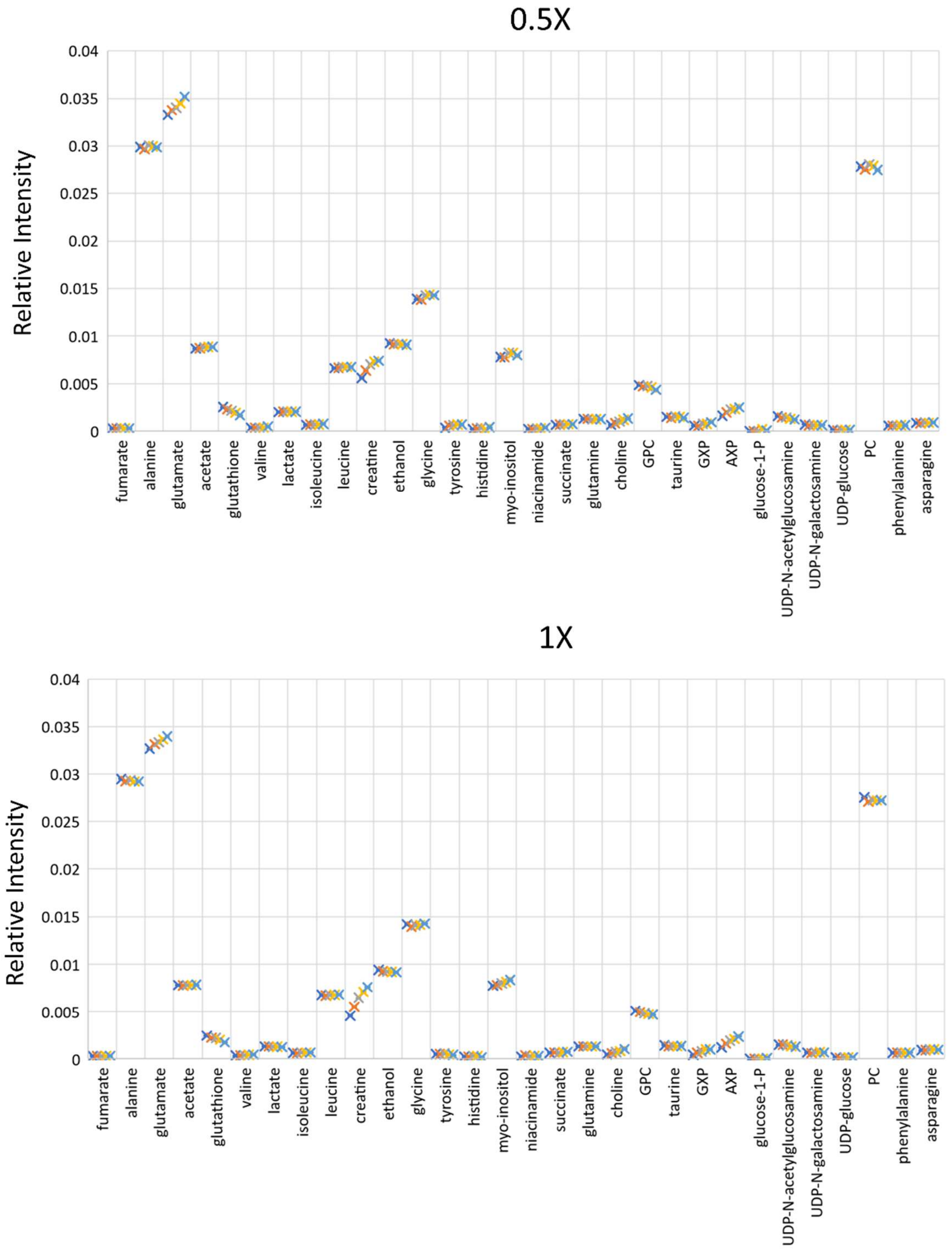

Figure S1. Stability test. Relative concentration levels of metabolites in a cell lysate sample, prepare with different concentrations of protease and phosphate inhibitor cocktail (0.5X and 1X) and acquired at different time points. Blue crosses: T0 , orange crosses: T=30 min, gray crosses: T=60 min, yellow crosses: T= 90 min and cyan crosses: T=120 min.

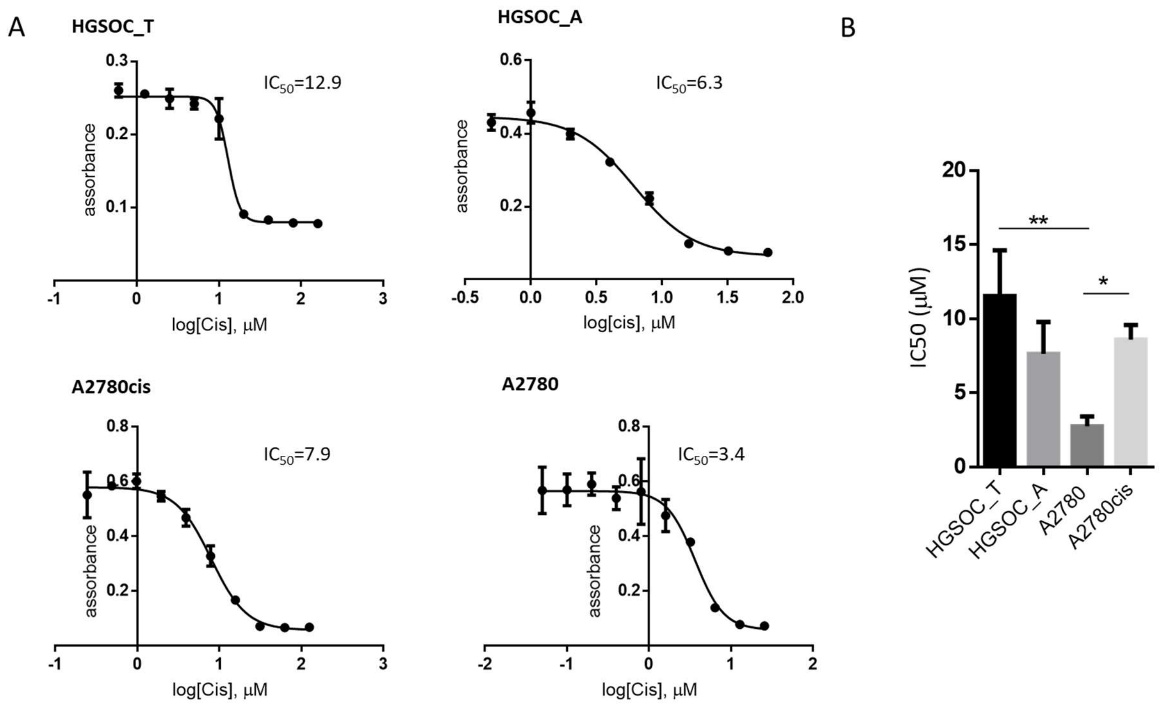

Figure S2. A) Representative cytotoxicity-curves of cisplatin after 72 hours of treatments determined by MTT assay. B) Histogram reports media±SD of IC_{50} measured in HGSOC_T , HGSOC_A, A2780 and A2780cis cells. Statistical analysis was performed with Anova using GraphPad Prism software; * indicates p-value < 0.05. **indicates p-value < 0.01
